# IMAGINE Personalities: Augmenting Digital Character Workflows Using Motion Capture, Wearable Sensors, and Live Coding

**DOI:** 10.3390/s25226976

**Published:** 2025-11-14

**Authors:** Dimitris Baltas, Anthie Kolokotroni, Katerina Malisova, Marina Stergiou, Giorgos Nikopoulos, Vilelmini Kalampratsidou, Alexandros Zarmakoupis, Martin Carle, Katerina El-Raheb, Iannis Zannos, Lori Kougioumtzian, Anastasios Theodoropoulos, Panagiotis Kyriakoulakos, Modestos Stavrakis, Spyros Vosinakis

**Affiliations:** 1 Department of Product and Systems Design, University of the Aegean, 841 00 Syros, Greecepank@aegean.gr (P.K.); spyrosv@aegean.gr (S.V.); 2 Department of Audio and Visual Arts, Ionian University, 491 00 Corfu, Greece; 3 Department of Performing and Digital Arts, University of the Peloponnese, 211 00 Nafplio, Greecek.elraheb@uop.gr (K.E.-R.);

**Keywords:** motion capture, wearable sensors, live coding, digital characters, personality expression, interactive media, transmedia workflows, animation, games

## Abstract

This study examines how emerging sensor-based technologies can augment the personality expression of digital characters across multiple media. While digital animation and games have traditionally relied on movement to convey traits, the integration of motion capture, wearable biosensors, and live coding introduces new opportunities for dynamic, embodied character design. Drawing on the MONOLOVE saga, we developed four prototypes across animation, games, interactive performance, and interactive networked environments. Central to our approach is the Wheel of Personality model, a structured taxonomy that organizes expressive parameters into four categories: Character Structure, Motion–Action, Interaction, and Environment. Each prototype was designed to explore how these categories, mediated through sensor technologies, contribute to the perception of personality traits. An evaluation with 14 participants from diverse backgrounds employed questionnaires and interviews to assess the alignment between intended and perceived character traits. The results show that movement and interaction were consistently identified as the most influential cues, while the impact of environmental factors varied across media. Additional influences included narration and the personality of the audience, underscoring the interpretive nature of perception. We conclude that personality expression emerges from the interplay of multimodal cues and context, offering methodological insights and frameworks for designing expressive and emotionally resonant digital characters in trans-media productions.

## 1. Introduction

Integrating sensors into the process of generating expressive digital characters has been applied for decades. Such workflows not only make animation creation more automated and time- and cost-effective, but also enable the design and development of digital characters with rich non-verbal nuanced expression through movement, gestures, and postures of human actors. As analyzed in previous work [1], the integration of both physical and virtual sensors can augment a digital character’s personality and behavior by making movement more fluid and interactions more dynamic. This allows for real-time adaptation, unconventional mappings, and new opportunities for creative experimentation, for example, by visualizing internal thoughts or behaviors as metaphors. These innovative workflows bridge the art of 3D digital animation with other arts and media such as performing arts, interactive media, games, and sound design. Each discipline comes with its own aesthetics, tools, workflows, technical challenges, and expressive codes.

The expressive behavior of virtual characters is foundational for believable interactive systems. Personality strongly influences how users perceive, interpret, and engage with narrative media, supporting immersion, social presence, and usability [2]. Research has shown that the effectiveness of digital characters depends not only on technical fidelity but also on their capacity to embody coherent and recognizable personality traits [3,4,5]. Classical animation principles provide strategies for expressive motion, but recent work has integrated frameworks such as Laban Movement Analysis to extend character believability through structured variations in gesture and dynamics [6,7]. Similarly, empirical studies reveal that multimodal cues—including body movement, facial expression, voice, and gesture—are correlated and jointly inform personality perception [8,9]. Recent developments in inertial sensor-based interfaces further enhance expressive character control through gesture recognition and dynamic mapping. Patil et al. [10] demonstrated that six-degrees-of-freedom inertial motion sensors can enable the expressive control of 3D avatars by recognizing gesture variations and synthesizing stylistic motion in real time, providing a low-cost and intuitive alternative to optical motion capture systems.

Wearable sensing technologies—including inertial measurement units (IMUs), biosensors (e.g., heart rate, galvanic skin response), and motion trackers—have matured to provide the continuous capture of both motion and internal physiological state. A recent systematic review of wearable systems in musical contexts highlights how inertial and physiological wearables have been used for gesture recognition, physiological monitoring, performance feedback, and sensory mapping—illustrating both the methodological opportunities and recurrent issues around comfort, calibration, and usability [11]. Frameworks combining low-cost motion capture with physiological or movement metrics have been used to infer stable personality traits or momentary affect in individuals. Delgado-Gomez et al. [2] is one such example, where joint movement patterns captured via Kinect were used to predict traits from the OCEAN personality model.

Although these technologies have been used to measure and even predict human personality, their application to digital characters remains limited. This work addresses that gap by researching the personality traits of digital characters in four different media—interactive media, animation, games, and interactive networked performances—and how they can be augmented through three sensory technologies: motion capture, wearables, and live coding.

This study is part of a broader research program on character personality in trans-media productions, conducted under the IMAGINE-MOCAP project. The project brings together a multidisciplinary team including animation directors, choreographers, performers, interactive media designers, game designers, sound artists, and developers. Its aim is to investigate how emerging sensor and live coding technologies can be used to enrich character personality. Specifically, we incorporate real-time motion capture and interactions, embodied reactions through biosensors, and live-coded audio–visual effects to address the research question of how these aspects contribute to the creation of characters with enriched personality behaviors.

In this work, we contribute to the field of sensor-based animation and embodied character design by

1. Proposing a workflow and framework that analyze the different modalities of a digital character’s personality across media.
2. Designing and developing four demos to test the unique opportunities and challenges of each medium in expressing personality traits.
3. Evaluating the workflow and framework through the audience perception of the developed characters.

Rather than treating the four media separately, we approach them together through a trans-media project, examining how the same digital character’s personality manifests differently across contexts. This allows us to study character personality through a transdisciplinary lens, deepening our understanding of its nuances and expanding its manifestations through embodied behaviors and abstract audio–visual metaphors, rather than through cartoon-like simplifications.

Our methodology combines motion capture techniques, live coding, and wearable devices to explore how digital character personality can be augmented across the four media. In interactive performances, motion capture suits and biosensor wristbands measuring heartbeat and skin conductance were used to control the avatar and its environment. For the animation, an actor performed under motion capture following creative direction to express the desired personality traits. In the game experiment, motion capture was used to record the digital character’s movements, focusing on interactions with objects and other characters. Finally, in the interactive networked environment, the creative team applied motion capture and live coding to translate performer movement into sound, reflecting the personality of a non-human character.

These methods were implemented in four demo applications, where we examined how a character’s personality traits differentiated across media. The demos were then tested and evaluated by audiences in the creative arts. The evaluation sought to determine whether the audience perceived (a) the intended personality traits, (b) the means used by the creative team to achieve them, as well as how perception differed across media.

The following sections present our approach to character personality in the four IMAGINE media. We then describe the methodology used to create the demos, from script development to the tools and methods applied to extract and project character personality. Finally, we discuss the evaluation results and outline directions for future work.

## 2. Personality in IMAGINE Media

The issue of a character’s personality in IMAGINE media is a recurring subject in research. The following is a selection of works that deal with personality in all of the four different media that were mentioned before, most of the time using sensor modalities of motion captures and wearables, or live coding technology. In interactive media performances, the use of motion capture techniques and wearable technologies have given artists the artistic freedom and the possibility to use the actors’ physical state and project personality characteristics in the digital world. Such an example is Cai et al. [12], who explored augmenting personalized systems with human-like characteristics such as behavior, emotion, and personality using rich personal data from information systems and ubiquitous devices like wearables.

In animation films, there is a significant number of studies about the use of stereotypes, specifically in relation to gender or age. In such a case, Gonzalez et al. [13] conducted a content analysis of popular children’s films and highlighted the strong associations between physical appearance, social attributes, and gender underscoring how they affect children’s body image and gender development. Shehatta [14] explored gender representations in the film Brave, showing how linguistics and visual elements combine to break traditional portrayals of female protagonists. Robinson et al. [15] on the other hand focused on the portrayal of older characters in Disney films and Bazzini et al. [16] examined the ‘what-is-beautiful-is-good’ stereotype in Disney movies.

In the games industry, the use of mainly motion capture and wearable technologies is usually preferred for personality trait expression. In “Cyberpunk 2077”, optical motion capture systems and inertial suits are used for more dynamic and expansive motion capture needs. In video games, motion capture is crucial for creating life-like characters that players can connect to. Games like “The Last of Us” and “Uncharted” use mocap to deliver emotionally engaging performances that enhance the storytelling and character development. Although wearables are not very commonly used in the games industry, there are some cases, like Nelepa et al. [17], that experiment with the use of wristbands to detect changes in affective states of players during gameplay. Brooks [18] explores the use of sensor-based systems that capture human input and map it to digital content, such as games and virtual reality, to create empowering, creative, and playful experiences. Similarly, Magar et al. [19] demonstrated that immersive virtual reality role-playing with virtual humans can enhance empathy and embodiment, highlighting the potential of VR-based interaction for emotionally responsive and personality-driven gameplay experiences.

Interactive networked environments are the most common aspect of artistic research where live coding can use its full potential. In combination with motion capture suits and wearable devices, they can enhance the sense of liveness for performance audiences. Kate Sicchio, in the performance “Sound Choreographer <> Body Code” uses code not only to generate music, but also to interact with dancers, creating a dynamic feedback loop, where choreography and coding influence each other in real time.

## 3. Materials and Methods

In this section we present all materials and methods that were used in this study. We start with presenting the Wheel Model, a custom model for collecting elements that can assist with designing digital characters. Then we proceed with the design requirements extraction for the implementation of the four demos that belong to each media. Afterwards we present each demo in detail in terms of its storyline, the technologies, the characters, and the intended personality assigned.

**Figure 1:**
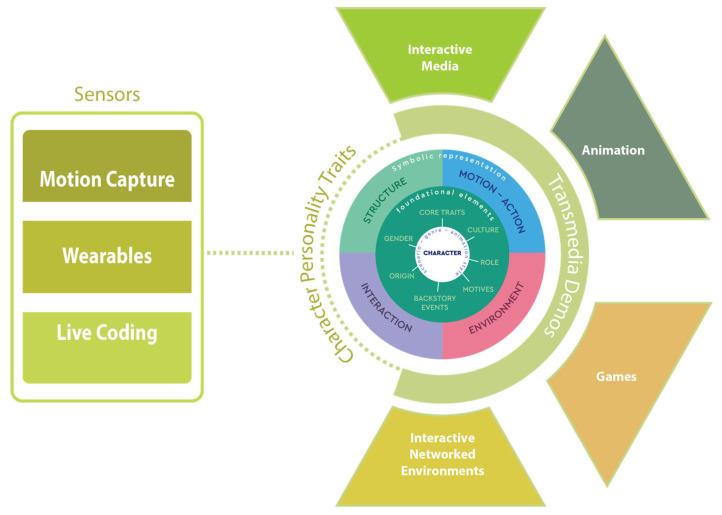
Methodology diagram.

Finally, we conclude with our evaluation methodology and the instruments used. An overview of the methodological workflow is illustrated below (Figure 1), highlighting the interrelation of sensor technologies and presentation medium in characters’ personalities, filtered through the Wheel Model.

### 3.1. The Wheel Model for Expressing Character’s Personality

**Figure 2:**
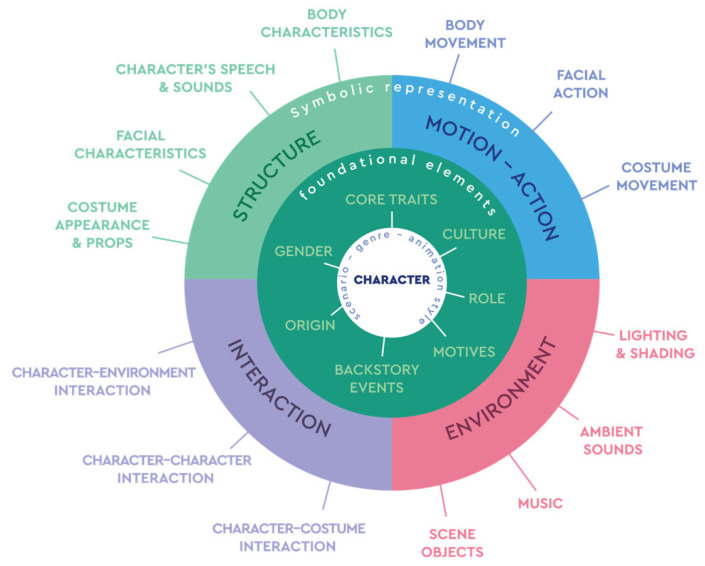
The Wheel of Personality Model—taxonomy of structural, performative, environmental, and interactive elements contributing to character personality.

The Wheel Model of Personality (Figure 2) is a proposed taxonomy that systematizes how digital characters convey personality across different modalities. It was developed during the initial stages of problem definition in the IMAGINE MOCAP project. Building on insights from psychology, animation, and game studies, it integrates findings on how shapes, colors, motion, sound, and interaction influence perception [20,21,22].

At its core, the Wheel of Personality organizes the diverse parameters that contribute to character personality into a structured taxonomy. The inner layer anchors the character in narrative and cultural context, defined by designer-led determinants such as scenario, genre, animation style, cultural background, role, motives, and backstory. Surrounding this core there are four primary categories of symbolic representations that articulate personality through observable features:

- Character Structure: encompassing anthropomorphism, proportions, shape, color, attractiveness, and costumes, which establish the character’s visual identity.
- Motion–Action: including gestures, postures, hand and facial motions, and overall movement dynamics, which reveal traits such as extroversion, confidence, or timidity.
- Environment: spanning lighting, shading, spatial design, and ambient sound, which situate the character and shape audience perception of personality.
- Interaction: covering speech, sound design, and behavioral patterns toward others and the environment, which further refine the character’s perceived traits.

The Wheel Model emphasizes that personality is not expressed through isolated elements but through the interplay of these categories. For example, research shows that personality expression often depends on combinations of cues rather than single elements. A triangular silhouette paired with sharp movements and low lighting typically signals antagonism or menace [23,24,25] A rounded body shape combined with warm colors, fluid gestures, and melodic vocal qualities conveys friendliness and approachability [22,26,27] A character with exaggerated head-to-body proportions, soft fabrics, and bright environments evokes vulnerability and innocence, often associated with childlike figures [28,29,30]. In contrast, rigid postures, metallic costumes, and fragmented or dim lighting create impressions of authority, tension, or emotional distance [31,32].

The value of the Wheel Model lies in translating creative and symbolic intentions into structured design decisions. By organizing personality-related features into a coherent model, it provides designers with a generative tool for aligning narrative goals with technical implementations. It aims to bridge semiotics and technology, ensuring characters maintain expressive coherence across diverse media such as animation, games, interactive media, and interactive networked environments.

In our methodology, the Wheel Model is operationalized in our methodology in three key ways:

(a) Workshops and expert interviews—as a conceptual guide for eliciting insights on how personality traits are communicated in design practice.
(b) Demos—to identify which personality-related elements were activated in prototypes and how they influenced audience perception.
(c) Evaluation—as a reference framework for systematically analyzing the expressive capacity and coherence of the designed characters.

### 3.2. Design Requirements Extraction

Personality expression has been an issue that creators in media, such as animation, games, interactive performances, and interactive networked environments, have been interested in for some time. Beyond technical fidelity, the question is how to design characters that feel authentic, expressive, and capable of evoking engagement through their traits, emotions, and actions. To ground the technological and methodological requirements of the project, the team conducted a combination of user workshops and expert interviews. These activities aimed to capture both practitioners’ perspectives and domain-specific expertise on what makes character personalities believable and expressive.

Two workshops were organized to gather input from practitioners. The first (Onassis Summer School, Athens 2024) focused on motion capture as a tool for personality expression, combining storytelling tasks with live performance in Rokoko suits. The second (CEEGS Conference, Nafplio 2024) invited participants to create characters in Unity using predefined avatars and environments, later embodying them via mocap performance. Questionnaire results from 13 respondents showed that participants prioritized body and facial characteristics, movement, and interaction as the most powerful cues for personality, while costumes, speech, or sound were viewed as less central. This highlighted the role of embodiment and non-verbal expressivity in character design.

In parallel, four expert interviews were carried out, each reflecting on personality in one of the IMAGINE media, with the following responses:

- The animation expert emphasized workflows built around Blender, Unity, and motion capture, while also grounding character personalities in cultural and historical archetypes. They noted that animation often treats characters symbolically, whereas games rely more on reusable archetypical movements and libraries.
- The interactive networked environments expert stressed real-time adaptability using sensors, OSC protocols, and live coding environments. They valued iterative pipelines where characters dynamically respond to performers, with personality seen as an evolving set of time-based traits.
- The games/networked environments expert discussed design processes involving Unity, RenPy, and Maya, balancing narrative with technical challenges such as VR motion sickness. They emphasized the need for custom animations where library assets could not capture individuality, while also drawing inspiration from titles like “The Last of Us Part II” where environment and pacing enrich personality portrayal.
- The interactive media expert favored open-source and flexible tools such as Godot for handling live data streams, with design priorities on dynamic responsiveness and interactivity, ensuring characters adapt in real time to performer or player input.

Together, these insights underline that expressive personality in digital characters requires a convergence of storytelling, cultural grounding, technical workflows, and real-time interactivity. The workshops and expert perspectives shaped the requirements framework of the project, directly informing the design of demo evaluations that test how different technologies can enhance the authenticity and adaptability of character personalities across media

### 3.3. Demos Implementation

As part of the evaluation framework, four prototype demonstrations were developed to investigate how personality traits can be expressed in digital characters across different media. Building on the workshops and expert insights, these demos served as experimental case studies for testing the taxonomy of symbolic elements (Motion–Action, Environment, Interaction, and Structure) in practice. Each prototype was designed to explore how personality and emotion can be conveyed through non-verbal communication, environmental design, and interaction dynamics. Together, they highlight complementary aspects of character expression.

For the purpose of the research, we decided to work with the story of MONOLOVE, by Giorgos Nikopoulos. The main characters of the story are two centaurs, representing a couple, but also the different aspects of a single person in the subconscious world. Ego, the male centaur, and Altero, the female centaur, are followed in a series of moments from their life both on the level of reality or in their imagination. In parts of the story, the character of a bird is also present, either as a single bird or as a flock of birds interacting with the two main characters in various ways, altering the relationship between them and acting as a trigger factor for some reactions.

In each medium, the characters transform into different versions of their own self, changing part of their appearance, but also projecting different personality aspects. For that reason, different technologies and methods were used to explore these aspects, according to each medium. In the following section, each demo is further analyzed and described in relation to the characters’ personality and the technologies used.

#### 3.3.1. Animation Demo

The prototype presented in this study is part of a short, animated film that explores the personality traits and emotional expression of digital characters through motion capture. This work demonstrates how the performances of actors/performers can be translated into non-human digital characters, specifically centaurs and birds, whose gestures, postures, and full-body movements embody personality traits and emotions. Rather than focusing solely on kinetic accuracy, the animation emphasizes the ability of motion capture to communicate personality through non-verbal communication, enhancing the narrative. The prototype scene presents a centaur with elongated, surreal legs reminiscent of Dali’s famous elephant struggling to balance on the sand, while a female centaur with the body of a turtle stands opposite. Also featured are some bird characters who act as protectors for the female centaur. Their contrasting forms emphasize fragility and grounded strength, embodied through motion capture to explore personality traits and emotions through non-verbal interaction (Table 1).

**Figure 3:**
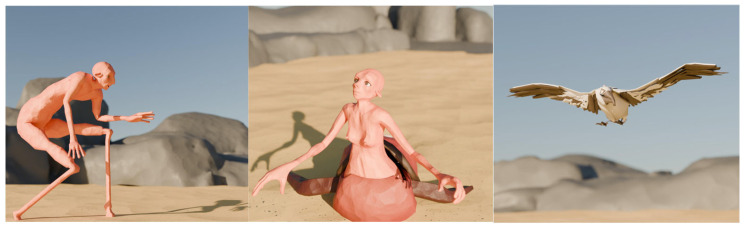
Animation demo screen captions.

The creation process followed a structured pre-production and production workflow that combined traditional filmmaking methods with digital animation. Initially, a decoupage was developed to outline the structure and sequence of shots, establishing the pace and rhythm of the narrative. Following this, a storyboard was created to illustrate key scenes, focusing on how body movement and gesture convey the storytelling without words. A mood board was then created, defining the overall aesthetic direction, color palettes, and atmospheric tone of the animated film. The next step was to create an animatic to test timing, editing, and spatial composition. This early visualization allowed for the choice of pace as the emphasis was placed on interpretation. The project then entered the animation analysis stage, where complex movements and expressive sequences were analyzed and segmented for efficient production. An analysis of the assets ensured that the character models, rigging, and prop elements were categorized and optimized for finalization. At the same time, environment design was carried out to place the animated performances within coherent spatial and atmospheric contexts that enhanced the narrative. As a result, we have the final animated image, where the motion data recorded was optimized and integrated into the characters. The project was completed with rendering, where visual enhancement, lighting, and post-processing techniques ensured cinematic quality. An important element of the project was the use of motion capture technology, specifically the RokokoSmartsuit Pro II, which captured full-body movements of the actors/performers. These movements were carefully designed to emphasize not only physical actions but also the embodiment of personality traits, such as self-confidence, insecurity, sensitivity, determination, and more. The captured movements were based on the storyboards and the script, ensuring that the expressiveness of the actors was faithfully translated into the animated centaurs and birds. This integration allowed the characters to transcend their non-human forms and appear emotionally authentic, demonstrating how motion capture can act as a bridge between human interpretation and digital character animation. The film was used as a test case to evaluate the personality traits and emotions of digital characters through audience empathy. Overall, the short, animated film served as an experimental prototype that highlighted the importance of non-verbal communication in storytelling, with a view to highlighting the personality traits of digital characters (Figure 3).

#### 3.3.2. Game Demo

**Figure 4:**
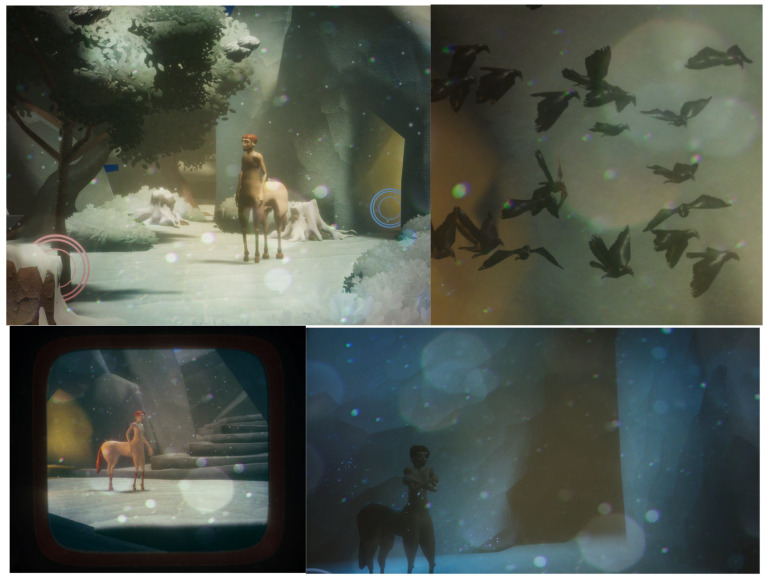
Game demo screens.

The game prototype is focused on one of the levels of the MONOLOVE game. Following the centaurs’ story in the animation film, the game explores the personality aspects of the two main characters—the male Ego and female Altero—in the context of an adventure puzzle game. The player follows the male centaur as it goes through a series of quests in order to connect and reunite with the female character. The demo only features the first level of the game, where the two centaurs are found in a cold unwelcoming cave. The player—with the form of the male centaur avatar—needs to find a way to make the cave a warm and cozy place. In order to do so, he needs to use his strength and brains and go through a quest inside and outside the cave to figure out a way to light a fire inside the room (Table 2 and Figure 4).

The design team used game designing methods to decide on the elements of the environment that could project the user’s personality and emotional state in each case. These decisions affected not only the look and feel of the game, but also the interactions of the character with other characters and objects of the environment. For the characters’ personality, the game design team used synthetic motion, based on the keyframe animation method. Also, for expressing different personality aspects, the prototype used various environment design parameters such as different lighting, objects, shades. The interaction of the character with objects and the environment was a strong element of personality expression as well. The design process also included the creation of a storyboard with the different steps of the hero’s journey, as well as an interaction mood board with the alternative story flows, according to the user’s choices. The image below shows a diagram of the game interactive narrative. For the look and feel of the game, the prototype used the style of the well-known 1990’s game of View-Master, with the signature shutter movement when changing camera views. Another aspect that the design process took into consideration was the way non-human characters can interact in the game for the benefit of the narrative, but also the personality expression. The motion of the flock of birds had to be studied and configured in a realistic yet expressive way to give the proper feeling to the users, but also guide them with subtle hints on the path to follow to complete the task. Various game design features were also used, such as physics and lighting effects, to create an environment that stands on the edge of realism but also to make the main characters and their actions and feelings stand out.

The game demo, focusing mainly on the personality traits and how they can project the digital characters’ emotional state, works in a supplementary way to the rest of the demos and the use of personality traits in an intermedia production, focusing and bringing to light different aspects of the character that the rest of the productions might miss. In the table below, the aspects of the Wheel personality that were used mainly in the game prototype are highlighted, giving a better view on the different personality factors that the game focuses on.

#### 3.3.3. Interactive Performance Demo

An interactive performance demo is a short experimental intermedia performance which lasts approximately 5 min and brings together live performers, digital environments, and real-time interactive technologies. It features two on-site performers, a live director/camera operator, a prerecorded narrator, and a projected digital environment on a large screen.

The performance is accompanied by a recorded narration that guides users through the plot. The two centaurs, the male and female, are sleeping in their cave. The male centaur is having a dream. The narrator explains the plot while the audience is following the live action.

One performer wears a Rokoko motion capture suit, directly animating in real time the avatar on screen—Dali’s centaur. The second performer wears a Shimmer wearable device on her wrist; her biometric signals (heartbeat and skin conductance) indirectly shape the digital environment projected on screen, influencing both visual and auditory elements. The prerecorded voice of the narrator (performed by the scriptwriter and actor of MONOLOVE) provides the narrative backbone, guiding the interplay among five distinct agents: the digital avatar (Dali’s centaur), animated in real time; the narrator’s voice, which exists only as sound; the motion capture performer, visible both on stage and through the avatar she controls; the biosignal performer, whose physiological data generate changes in the digital environment, visible on screen as dynamic visual effects and sounds; and the live director, who manages real-time camera control and technical operations within the Unity game engine (Table 3).

**Figure 5:**
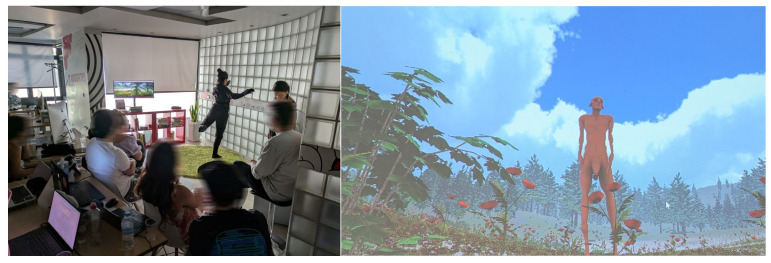
Interactive media demo.

Together, these elements create a hybrid performance that blurs the boundaries between live action, digital media, and immersive storytelling (Figure 5).

The Wheel aspects used in this media are body movement (through Rokoko), environment (objects/lighting and shading; through biosignals), voice (through narrator), and character–environment interaction. Below we present in detail the different parts of the performance:

Part 1: The performance starts. The motion capture performer is on stage. The recorded narrator begins playing in the background. The live director demonstrates the whole digital world to finally show the digital avatar, Dali’s centaur. The motion capture performer moves, and the digital avatar follows her movements. The narrator talks about “my body… I have half of my body… and I feel half as a creation. Only the space around me is full and complete… What keeps me is the body to come… the other half of the body.”

Part 2: At this point, the second performer, the biosignal performer, enters the stage. “Two centaurs are sleeping in their cave, hugging each other. The fire in the cave runs low…” At this point, the two performers act together to compose the scenery in the digital world; one defines the movements of the centaur, and the other one defines the sounds and visuals of the digital world.

Part 3: The biosignal performer leaves the stage, and the motion capture performer stays again alone on the stage. Narrator: “Now again, I have half of my complete body, and I feel half again as a creation. Only the space around me is perfect, united…”

#### 3.3.4. Interactive Networked Environment Demo

The interactive networked environment demo uses the bird characters of the MONOLOVE universe in a remote sound performance experiment using live coding and motion capture methods. The demo was recorded using two remote locations, in Athens and Corfu, where two separate teams were collaborating in real time to create a soundscape according to the characters’ personalities, expressed through the performer’s movements. For the live coding part, the creative team used the software SuperCollider(v.3.14.0), an open-source platform for audio synthesis and algorithmic composition, widely used in electronic music, sound art, and live coding performances. Through its programming language it allows users to design instruments and generate complex soundscapes but also control them interactively. For the motion capture part, the performer was using a Rokoko suit that gave access to the live-coding team to the performer’s movements in real time.

**Figure 6:**
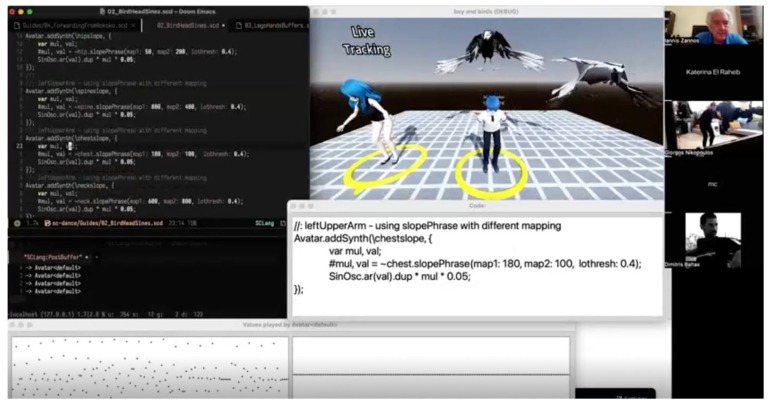
Interactive networked environment demo screenshot.

During the demo, the performer, located in a studio in Athens, was moving and animating the bird character from the MONOLOVE script (Figure 6 and Table 4). The team in Corfu was receiving the raw movement data in their studio, and, through their system, translating it to different sounds. The performer listening to the soundscape could modify his moves, the intensity and range of motion, to create the desired sounds. The whole demo was also broadcasted to audience members, through Zoom conference software(v. 6.5), in different locations across the world. The demo consisted of three parts, where in each part the creative team was changing the sound, but also the part of the performer’s body that would control the soundscape, showing part of the potential of the system, and the possibility to create a much more complex soundscape when using all 19 joints that the Rokoko system can transmit.

### 3.4. Sensors and Taxonomy Wheel Connection

The integration of motion capture and physiological sensing technologies in the demos offers a multidimensional approach to shaping and expressing personality in digital characters. Each sensor modality contributes to different facets of expressivity by translating internal bodily states into perceivable visual, auditory, or behavioral changes. These contributions can be organized around the four interrelated dimensions that define the taxonomy Wheel.

Motion-related sensors, such as motion capture systems and inertial measurement units (IMUs), provide high-resolution data on body posture, gesture, and dynamics. These parameters influence the Motion–Action and Interaction aspects of character behavior, enabling the representation of traits such as extraversion, confidence, or hesitation through movement amplitude, tempo, and rhythm. For the four demos, motion-related sensors are used in most of the demos, mainly in animation, interactive networked environment, and interactive media performance, through motion capture suits.

Physiological sensors, including electrocardiogram (ECG), electrodermal activity (EDA), electromyography (EMG), skin temperature, and respiration sensors, link internal affective states with outward expression. Variations in heart rate, galvanic skin response, or muscle tension can modulate both the Structure (for instance, skin tone or micro-expressions) and the Environment (such as lighting, sound, or color intensity) to externalize emotional states such as calmness, excitement, or anxiety. Physiological sensors in the demos were mainly used in the interactive media performance.

Thermal and respiratory measurements provide additional cues about emotional regulation and arousal. Changes in temperature and breathing rate can serve as subtle indicators of stress, relaxation, or emotional intensity, further enriching the coherence between physical and digital embodiment.

Wearable and networked sensor systems extend these principles into interactive contexts, where the performer’s real-time data directly influence character or environmental behavior. This real-time coupling enhances the Interaction dimension, enabling adaptive expressions of personality that respond dynamically to context and audience. This type of sensor, together with thermal and respiratory sensors, were also used in the interactive media performance.

Finally, sonification and audio mapping introduce a crossmodal layer of expression by transforming biosignals and motion data into sound parameters. These auditory correlations augment the Environment and Interaction dimensions, reinforcing empathy and emotional resonance between performer, character, and observer. These sensors were used in the interactive networked environment demo.

Overall, the combined use of motion and physiological sensors enables a holistic translation of embodied data into expressive traits. By synchronizing bodily signals with the audio–visual attributes of a digital character, these systems foster a sense of emotional presence and coherence that supports the perception of personality as a living, responsive construct. In the table below (Table 5), each sensor type is associated with the specific component of the personality taxonomy it most strongly influences within the expressive framework.

### 3.5. Evaluation Methods

To sum up, in this work, we explore the concept of expressing digital characters’ personalities, across four different digital media (games, animation, interactive networked environments, and interactive media), inspired by MONOLOVE’s storyline and by utilizing sensory technologies like motion capture, wearables, and live coding. To do so we worked in the following way: Initially we studied the current literature and filmography, identifying how a character’s personality is currently expressed in various digital media. Afterwards, we shaped the Wheel Theoretical Model that is presented in detail in Section 3.1 and was used as a common language along the whole project. Afterwards, we conducted interviews (presented in Section 3.2) with four experts on the four digital media.

Prior to the development of the four media prototypes by our team, an important step was to determine how personality would be assigned to the digital characters of each, and of course, how that personality would be measured. A creative team behind each demo, consisting of various expertise like game developers, dancers, wearable specialists, live coders, performers, designers, and animators, decided the elements of the Wheel Model that would be utilized for each media and also the storyline and the personality they would like to give to each character. Drawing inspiration on the theoretical frameworks of personality (personality models) presented earlier in this section, the teams used a custom personality trait model which is presented in detail in Section 3.5.3. Afterwards, the four creative teams prototyped the four demos.

#### 3.5.1. Evaluation Methodology

The next stage included the evaluation of each demo. The purpose of the evaluation was to test whether the intention of the creative teams in terms of the character’s personality was communicated to the audience, or in other words, if the audience perceived the same personality as the one described by the creative team. To proceed with such an evaluation, we invited 14 users that were asked to watch or interact with each of the prototypes, followed by interviews and questionnaires. The results of this study are presented in Section 4.

#### 3.5.2. Procedure

The evaluation followed a mixed-method approach. Questionnaires provided quantitative measures of perceived character personalities while also capturing qualitative insights regarding which design elements influenced participants’ judgments. Semi-structured interviews offered deeper qualitative exploration, allowing participants to articulate how specific expressive elements contributed to personality perception. The primary goal was to assess whether participants perceived the intended personality traits in each prototype and which expressive elements, as outlined in the Wheel Model, were most influential across different media.

The evaluation procedure was conducted across three separate sessions, each lasting approximately 60 min and involving a subset of participants, to accommodate participant availability and ensure a comfortable environment for interaction and discussion. This setup also facilitated detailed observation and manageable data collection.

Each session consisted of the following steps:

- Step 1: Demonstration Phase

Participants experienced all four prototypes in the same fixed order within each session. Each prototype lasted 1–10 min.

- Step 2: Questionnaire Phase

After each prototype, participants completed a short questionnaire (approx. 10 min) that assessed perceived personality traits using a custom trait list, derived from related models of personality, as well as adjectives provided by the narrators and creators of the story, capturing nuanced personality expressions unique to the characters. Participants rated each trait on a 0–5 Likert scale (0 = “not at all”, 5 = “very much”). They also indicated which of the Wheel Model elements contributed most to their perception of personality by importance, and could suggest additional elements to enhance personality expression.

- Step 3: Interview Phase

After all prototypes, participants engaged in a semi-structured interview (approx. 20 min) to discuss their impressions, differences across media, and alignment with the Wheel Model categories. This provided qualitative insights into the reasoning behind personality perception, allowing participants to elaborate on subtle design cues and expressive choices that influenced their judgments.

#### 3.5.3. Custom Traits List

The purpose of developing a custom trait list was to capture a set of qualities that the creators intended to express consistently across the four media prototypes—animation, games, interactive performance with wearables, and interactive performance with live coding. These traits served a dual role: first, as design anchors that guided how personality would be embodied within each medium, and second, as evaluation criteria in the questionnaires, where participants were asked to recognize and assess whether the intended traits were successfully conveyed by the characters.

A single framework such as the Big Five (OCEAN) [33] was not sufficient for our purposes. First, established frameworks like the Big Five are expressed in abstract psychological terms that do not always map directly to narrative design contexts. For example, “Conscientiousness” or “Openness” may be meaningful to psychologists, but are not readily interpretable by participants evaluating a fictional character’s behavior. Second, traits had to be narratively resonant, adaptable across expressive modalities, and intuitively understandable to non-experts. Thus, instead of applying a single model, we conducted a filtering process that combined the following:

1. Theory-grounding traits in validated psychological and expressive frameworks.
2. Creative insight-aligning traits with the story world of MONOLOVE (themes of vulnerability, identity, and transformation).
3. Practical design relevance-ensuring traits could be embodied through movement, sound, and interaction across diverse media.

This integrative approach led us to distill theoretical complexity into eight accessible, narratively anchored traits: Independent, Decisive, Self-confident, Strong, Sensitive, Weak, Fearful, and Aggressive.

To clarify how this list was derived, we next outline how different models contributed complementary perspectives.

The Big Five, or OCEAN model [33], describes personality in terms of five broad dimensions: Openness, Conscientiousness, Extraversion, Agreeableness, and Neuroticism. It is widely considered the most empirically supported trait framework and has been applied in digital character design to modulate behaviors such as speech, gesture, or responsiveness. In relation to our list, several traits map directly onto these dimensions. Independent and Decisive relate to Conscientiousness and Extraversion, representing autonomy, discipline, and assertive decision-making. Self-confident corresponds to low Neuroticism (emotional stability), while Strong reflects high Extraversion combined with emotional stability, embodied in resilience and dominance. On the other hand, Sensitive corresponds to higher Neuroticism and aspects of Agreeableness, reflecting vulnerability and empathy. Fearful links strongly to high Neuroticism, while Weak resonates with low Extraversion and Conscientiousness. Finally, Aggressive aligns with low Agreeableness, characterized by dominance and hostility. Thus, the Big Five provided a scientifically robust foundation for our custom list, though its broad dimensionality required refinement into more specific, narratively usable traits.

The MBTI [34] expands Jungian psychological types into 16 personality categories based on four dichotomies: Extraversion–Introversion, Sensing–Intuition, Thinking–Feeling, and Judging–Perceiving. Although criticized for its binary structure and lack of reliability [35], MBTI has proven narratively powerful by offering intuitive archetypes useful for storytelling. This archetypal clarity influenced our trait list. The Commander (ENTJ), characterized by decisiveness, confidence, and assertiveness, directly inspired traits such as Decisive and Strong. In contrast, the Mediator (INFP), defined by empathy, sensitivity, and introspection, informed traits such as Sensitive and Fearful. In this way, MBTI helped us frame oppositional contrasts central to MONOLOVE’s narrative (e.g., decisiveness vs. fear, strength vs. weakness). While MBTI’s scientific shortcomings meant it could not serve as the primary foundation, its role in inspiring archetypal contrasts was crucial in shaping our custom list.

The OCC model [36] classifies 22 emotions into categories linked to events (goal relevance), agents (moral/social evaluation), and objects (intrinsic preferences). It has been foundational in affective computing, providing a rule-based framework for generating consistent emotional responses. For our list, OCC informed traits that derive from appraisals of threat, vulnerability, and moral evaluation. Fearful maps directly onto event-based appraisals of threat or anticipated harm, while Sensitive corresponds to appraisals of others’ well-being and intrinsic preferences, emphasizing empathy and moral concern. Aggressive reflects agent-based evaluations of hostility or antagonism. Thus, OCC enriched our list by grounding traits in the “why” of emotions, connecting character behavior to appraisal-driven motivations.

The Realact model [37] integrates personality traits with emotion regulation in a hybrid framework that combines a continuous affective state manager with an event-based behavioral scheduler. It shows how traits such as Extraversion and Emotional Stability modulate expressive behaviors like posture, gaze, and gesture. For our list, Realact reinforced distinctions between traits associated with consistent, stable expression (e.g., Self-confident, Strong) and traits tied to emotional instability or over-modulation (e.g., Fearful, Aggressive). Realact’s emphasis on behavioral regulation highlighted how Weak could be expressed through subdued, inhibited behaviors, while Aggressive emerges through over-modulated, dominant actions.

The Communication Styles Inventory [38] identifies six communication styles: Expressiveness, Preciseness, Verbal Aggressiveness, Questioningness, Emotionality, and Impression Manipulativeness. It explains how personality and emotion manifest in speech and interaction. In our list, Aggressive links closely with Verbal Aggressiveness, while Sensitive reflects Emotionality, capturing vulnerability and affect-laden interaction. Decisive overlaps with Preciseness, emphasizing clarity and determination in communication. CSI thus provided a bridge between dispositional traits and linguistic/interactional expression in our framework.

Laban Movement Analysis [39] describes movement through four dimensions: Body, Effort, Shape, and Space. Within Effort, qualities such as bound vs. free flow or strong vs. light weight convey psychological and emotional states. This model was critical for traits expressed through embodiment. Strong corresponds to movements with strong weight and expansive space, while Weak is reflected in light, bound, and constrained movements. Fearful can be expressed through bound, hesitant movement, whereas Self-confident appears in free, expansive gestures. LMA therefore enriched our trait list by ensuring that each quality could be consistently embodied in physical performance, particularly in animation and interactive performance contexts.

In Table 6, we summarize the relationship between our custom traits and the theoretical models reviewed. Each row presents one of the eight traits—Independent, Decisive, Self-confident, Strong, Sensitive, Weak, Fearful, and Aggressive—alongside the models that informed it and its core interpretation, which was ultimately defined by the creative team to align with the narrative themes and expressive goals of the project.

## 4. Results

This section presents the findings of the evaluation of the four demos based on data collected from multiple sources. The results are organized into three parts. First, the demographic data of the participants are described in Section 4.1. This is followed by the analysis and recording of the results of the questionnaires administered in three demonstration sessions, which provide information on the participants’ perception of the digital characters’ personalities (Section 4.2). Finally, the results of the interviews conducted with the participants are reported, offering a more in-depth understanding of their experience and opinion on each of the four demos (Section 4.3).

### 4.1. Participants

The evaluation involved 14 participants aged between 27 and 42 years, recruited from university communities, research centers, and creative networks, with no prior involvement in the prototypes. All participants reported moderate familiarity with at least one IMAGINE medium, but had not previously encountered the MONOLOVE storyline. The group represented a diverse range of professional backgrounds, including game design and development, industrial design, digital design, music composition, acting, choreography, directing, law, commerce, and digital storytelling.

This diversity was intentional, as it provided a broader spectrum of interpretive perspectives, ensuring that the evaluation captured how personality expression is perceived by audiences with different creative, analytical, and narrative sensibilities. Participants with technical or design experience could assess the intentional use of expressive elements, while those from artistic or narrative-oriented fields offered insights into emotional and symbolic interpretations. This interdisciplinary mix aligns with the nature of IMAGINE media, which integrates aesthetics, interaction, and narrative design.

The selection criteria included an interest in digital media and interactive experiences and no prior exposure to the prototypes to avoid bias. All participants within each session experienced the four prototypes in the same fixed order for consistency across the group.

### 4.2. Questionnaires

Below, we present some of the key findings from the questionnaires. To analyze whether the audience identified the Wheel elements and perceived the personality traits, we focused only on responses rated 4 or 5 on a Likert scale from 0 to 5, as these indicate a strong level of agreement or recognition. In addition to these findings, we also present other noteworthy results

#### 4.2.1. Animation

For Dali’s centaur, who was described as sensitive, fearful and weak by the designer, out of 14 participants only 35.7% described him as Sensitive, 42.9% as Weak, and 57.1% as Fearful.

Moreover, 35.7% rated the male character as Independent, 28.6% as Decisive, 28.6% as Confident, 14.3% as Strong, and 0% as Aggressive.

The female character was described as independent, decisive, confident, and strong. Out of 14 participants, 50.0% rated the female character as Independent, 28.6% as Decisive, 28.6% as Confident, and 50.0% as strong. The other traits were rated as follows: 42.9% as Sensitive, 21.4% as Weak, 7.1% as Fearful, and 0% as Aggressive.

**Figure 7:**
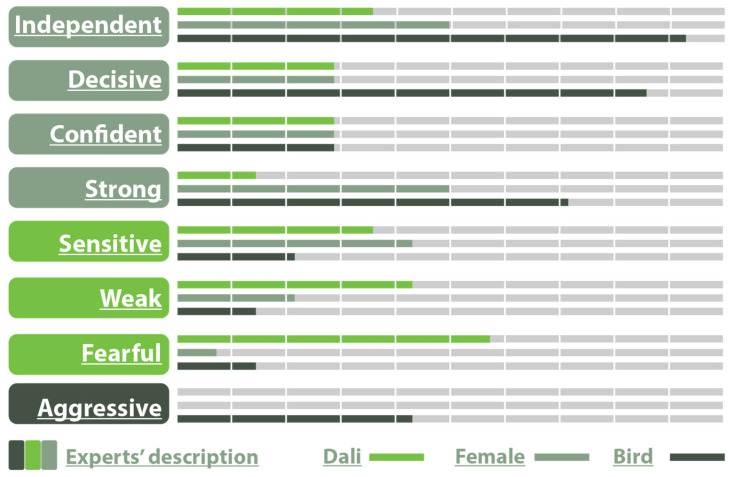
Animation characters’ personalities evaluated by participants.

For the bird character, 92.9% rated the birds as Independent, 85.7% as Decisive, 28.6% as Confident, 71.4% as Strong, 21.4% as Sensitive, 14.3% as Weak, 14.3% as Fearful, and 42.9% as Aggressive which was the intended trait (Figure 7).

The elements of the Wheel Model utilized for the animation demo according to the participants were body movement (100%), character–character interaction (85.7%), body characteristics (71.4%), and face characteristics (71.4%). However, scene objects scored below average, (42.9%), while lightning and shading were not considered to contribute much (35.7%).

The above answers were also confirmed by the answers to the question “Which of the four basic categories of the wheel do you think is the most important for Animation media”, where Movement scored 92.9%, followed by Interaction (85.7%), Structure (71.4%), and lastly Environment (21.4%).

Some interesting responses to the open question “Would you use another element/tool that has not been utilized?” were as follows: “The character’s gaze”, “the focus/direction of the camera”, “some background story about the characters”, “Music”, and “conversation between characters”.

#### 4.2.2. Game

In the game media, the male centaur was considered to be independent, decisive, and confident by the designer. Indeed, the audience thought this character to be Independent (64.3%), Decisive (85.7%), Confident (78.6%), Strong (50.0%), Sensitive (35.7%), Weak (7.1%), Fearful (7.1%), and Aggressive 0%, which confirms the designer’s intentions.

The female character was designed as sensitive, fearful, and weak. The audience thought of her as Independent (0.0%), Decisive (0.0%), Confident (7.1%), Strong (0.0%), Sensitive (57.1%), Weak (42.9%), Fearful (64.3%), and Aggressive (0.0%) which generally aligns with the designer’s intention if we exclude Weak which scored slightly below average.

**Figure 8:**
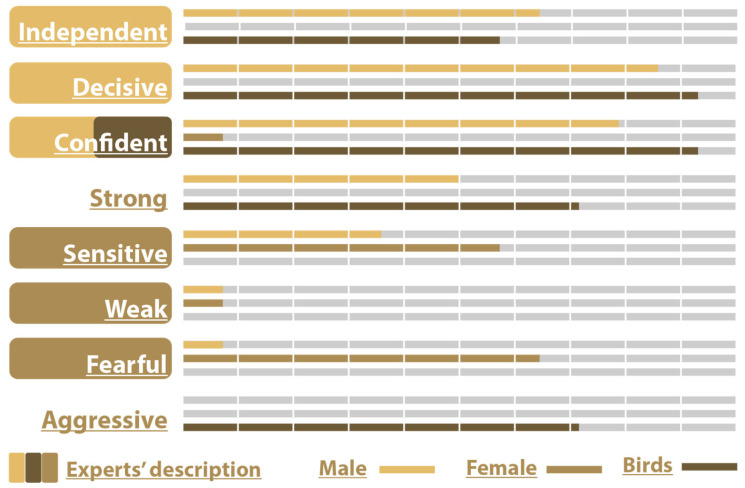
Game characters’ personalities evaluated by participants.

The birds’ intended confidence was confirmed by the audience with a score of 92.9%. The other characteristics were Independent (57.1%), Decisive (92.9%), Confident (92.9%), Strong (71.4%), Sensitive (0.0%), Weak (0.0%), Fearful (0.0%), and Aggressive (71.4%) (Figure 8).

The elements of the Wheel utilized for the Game demo according to the participants were body movement (85.7%), character–character interaction (78.6%), body characteristics (78.6%), and face characteristics (50%). Also, scene objects scored 50%, while lightning and shading scored 64.3% and character–environment interaction scored 64.3%. So, all Wheel Model elements used by the designer were successfully recognized by the audience.

The answers to the question “Which of the four basic categories of the wheel do you think is the most important for Game media” yielded the following responses: Interaction (85.7%), Movement (78.6%), Structure (57.1%), and Environment (50%).

Finally, the responses to the open question “Would you use another element/tool that has not been utilized?” included the following: “the focus/direction of the camera”, “storytelling”, “Music”, and “conversation between characters”.

#### 4.2.3. Interactive Network Environment

As for the two birds, out of 14 participants, 57.1% described them as Independent, Confident, and Strong, and 50.0% as Decisive.

**Figure 9:**
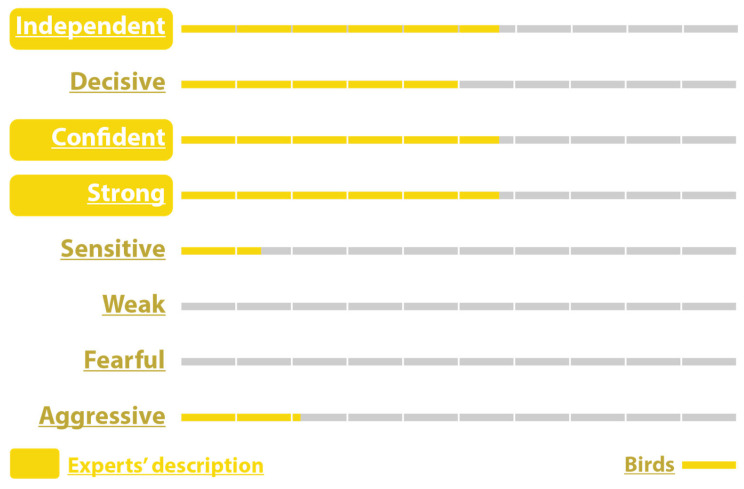
Interactive networked environment character’s personality evaluated by participants.

On the other hand, only 21.4% responded with Aggressive, 14.3% considered them Sensitive, and no one rated them as Weak or Fearful (0%) (Figure 9).

The elements of the Wheel utilized for the interactive network environment demo according to the participants were associated with the expression of the birds’ personality and were body movement (100%), body characteristics (57.1%), face characteristics (28.6%), and character speech and sounds (42.9%). Also, environmental sounds and music (64.3%) captured a high percentage of audience perception.

The answers to the question “Which of the four basic categories of the wheel do you think is the most important for interactive network environment media” were Movement (100%), Environment and Structure (42.9%), and Interaction (7.1%).

Finally, the responses to the open question “Would you use another element/tool that has not been utilized?” included the following: “360-degree field of view, interaction in a virtual realistic environment, less realistic rendering of facial and body characteristics”.

#### 4.2.4. Interactive Media Performance

**Figure 10:**
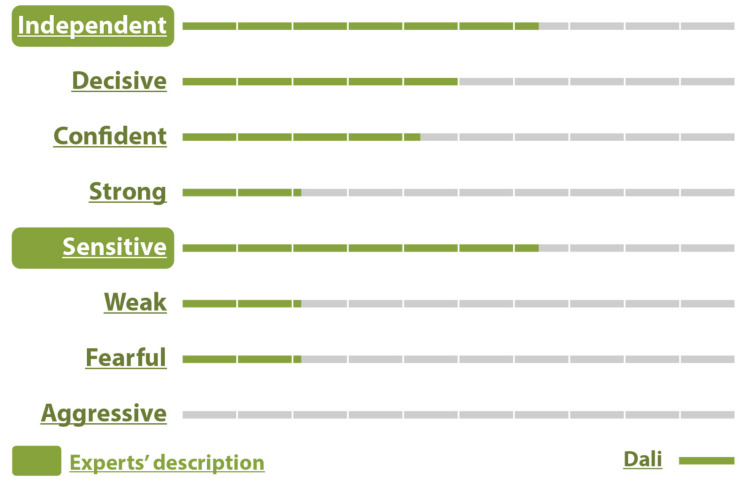
Interactive perofrmance character’s personality evaluated by participants.

As for the male centaur—Dali character, out of 14 participants, 64.3% described him as Independent and Sensitive, 50.0% as Decisive, and 42.9% as Self-confident. Also, 21.4% of participants rated this character as Strong, Weak, and Fearful, which means that the character confused them, and no one thought he was Aggressive (0%) (Figure 10).

The elements that contributed to the expression of the male centaur’s personality according to the participants were body movement (92.9%), character–character interaction (78.6%), environmental sounds and music (71.4%), and scene objects (57.1%).

Also, body characteristics (64.3%), face characteristics (42.9%), speech and sounds (42.9%), appearance (42.9%), and lighting and shading (50%) were recognized.

The answers to the question “Which of the four basic categories of the wheel do you think is the most important for Interactive Network Environment media” were Movement (92.9%), Environment (64.3), Structure (57.1%), and Interaction (42.9%).

For the question “Do you consider the performers’ characters within the performance?”, the participants answered ‘YES’ (78.6%) and ‘NO’ (21.4%).

The participants considered the role of the performers in the interactive performance to be important. More specifically one participant said that he really liked the, consciously- and unconsciously made, comparison on how he perceives what the performer is doing and what they see on the screen. Another said that they were very natural in the space, and some others said that they gave life and emotion to the digital character since they created the sound and movement.

One participant felt that the digital character was one with the performer, while another said that the character’s body movement was unique and attached to the performers.

In addition, one participant considered that there was a direct connection between the characters to the work through movement, heartbeats, and other means of connection and that the movements had an immediate response. Someone else separated the performers and observed that one performer identified two of the elements she assessed as most important, movement and interaction with the environment, while for the second performer he was not sure if she fully understood that she was participating beyond the movement of the river waters.

Another agreed that they directed the sounds, so this makes them part of the environment, and someone else added that their movements were part of the final result (through the avatar) and strongly affect the viewer’s experience.

Finally, one participant considered that although the performers perform in a live-action format, the final character is the union of the avatar with the performer’s movement and interpretation and not themselves on their own.

When asked if they would use any other element (visual, audio, interactive, etc.) to convey the personality of the characters that has not already been used in the performance, half of the participants answered that they did not miss anything while the other half said there would be some things that could enhance the performance.

One participant said that he would like to see the triptych “motive–history–personality” while some others said that it would help to show the character’s facial characteristics more.

One participant missed the character’s speech while another missed the lighting. On the other hand, one participant would like a less realistic depiction of the body type/face while another would like better connection and communication of selected media, e.g., the interaction of movement–sound–image.

When asked what changes they observed and to which elements of the digital world (e.g., environment, sound, image, movement) they believed they corresponded, most perceived changes in the landscape, weather, and natural elements in the virtual environment. One participant said that he observed the water of the lake overflowing and the flowers waving in the wind and believed that the movement of the flowers came from the performer’s breathing while that of the lake from the bracelet, while some others agreed that there were real changes in the buoyancy of the lake water, the appearance of some graphics (flowers), and a change in the sounds of the environment (wind intensity) and ground movement.

Someone else observed changes in the water, the lake, the grass, and plants on the ground in the light, but considered that they did not happen organically, as if someone was giving orders and considered that it was not a natural consequence of what was happening in the performance. Many said that they noticed changes in the sound and image and that these changes contributed significantly to the character’s personality and the story. Finally, one participant noted that there was a change in emotions which enhanced the narrative.

The biometrics related to the changes in the digital world, according to the 14 participants, were movement (100%) and cardiac activity (64.3%), which were recognized as the most significant. Additionally, sweating (35.7%), breathing (35.7%), and body temperature (28.6%) were also associated with the changes, though less frequently.

When asked how the changes perceived in the previous question were related to the character’s experience, the participants who perceived the changes considered them to be directly related. More specifically, one participant said that they influenced the psychology of the digital character and how positively or negatively he saw the events during the performance, while another noted that there was a strong interaction. One participant emphasized that the result seemed very alive and there was a connection, while another said that at the point where explosive movement was created (tension in the environment—the water rose, and tension—the sound of the wind) he felt the character’s “pain” more intensely, specifically he said “The performer’s movement in space as the camera turned, emphasized the beautiful environment and the pained character.” A participant observed that the performer’s movement contributed to the change in water level, her heart activity and breathing to the ambient sounds (wind sound), while her sweating contributed to the appearance of the graphics.

The elements that provided insights into the character’s personality according to the participants were performer’s movement (performer 1) (92.9%) and voice narration (71.4%), which were recognized as the strongest indicators.

In addition, biometric changes were linked to environment–sound (performer 2) (50%), camera movement, and direction (operator) (50%), and biometric changes linked to visual elements (performer 2) (35.7%) were also noted as contributing to the understanding of the character.

### 4.3. Data Quantitative Analysis

The evaluation results from the four demos were quantitatively analyzed using two complementary statistical approaches.

The first examined the consistency rate, defined as the percentage of personality traits intended by the design team that were correctly identified by at least 50% of the participants (N = 14).

The second analysis applied nonparametric statistical tests (Friedman’s and Wilcoxon’s) to determine whether the level of audience recognition differed significantly across the four evaluated demos (animation, game, interactive network environment, and interactive media performance).

#### 4.3.1. Consistency Rates Across Demos

**Figure 11:**
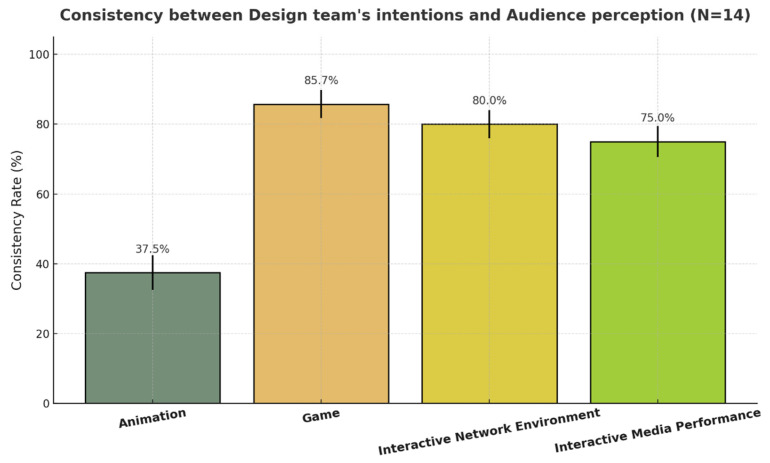
Consistency between design team’s intentions and audience perception.

The consistency rate results are presented in Figure 11: consistency between design team’s intentions and audience perception. Overall, the participants’ recognition of the intended expressive traits varied substantially across the different media. The game demo achieved the highest agreement with designer intentions (M = 85.7%), followed by the interactive network environment (M = 80.0%) and the interactive media performance (M = 75.0%). The animation demo showed a notably lower consistency (M = 37.5%).

Figure 11 displays the mean consistency rates across the four demos with standard deviation error bars. The game demo stands out as the most consistent, showing minimal variability across participants, while animation demonstrates a clear decline in recognition success.

#### 4.3.2. Friedman’s Test and Wilcoxon’s Signed-Rank Tests

We used the Friedman test to examine whether audience recognition of intended character personality traits differed across the four media conditions.

A significant main effect was observed, χ^2^(3) = 36.09, *p* < 0.001, indicating that the level of recognition varied substantially by medium. Post hoc Wilcoxon’s signed-rank tests revealed that the animation demo yielded a significantly lower consistency than all other conditions (*p* < 0.001), while the game demo achieved significantly higher scores than both the interactive network environment (*p* < 0.001) and interactive media performance (*p* < 0.001). No significant difference was observed between the interactive network environment and interactive media performance (*p* = 0.81).

These results demonstrate that interactivity and embodied engagement strongly enhance the communication and recognition of expressive personality cues in digital characters.

**Figure 12:**
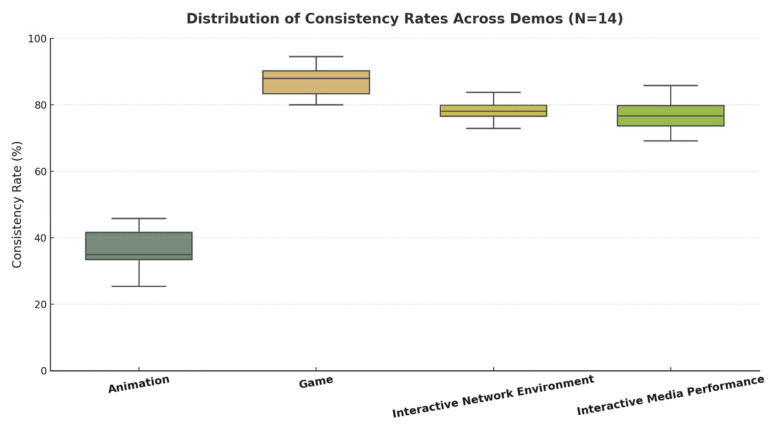
Distribution of consistency rates across demos.

Figure 12 further illustrates the distribution of participant responses using boxplots. Each box represents the interquartile range (IQR) and median values, while individual dots indicate participant-level scores. The animation condition shows both the lowest median and limited spread, confirming its weaker communicative performance. Conversely, game presents a higher and more stable median consistency, reflecting strong alignment between expressive intent and perception. Interactive network environment and interactive media performance occupy a middle ground, with slightly greater variability due to their open-ended and embodied nature.

### 4.4. Interviews

The interviews were semi-structured. We followed the same questions for the three groups and, according to the answers, the conversation evolved.

When asked if they perceived any differences in the character of the centaur (man with tall legs) in the animation and the performance, one participant noted that the perception of the character was much clearer in the animation because the facial characteristics and expressions of the characters could be easily seen. The participant also added that the performers were surely expressive during the performance; however motion alone was not sufficient and facial characteristics were needed. This was also confirmed by two more participants, who said that when watching the performance, they paid attention to the moves and kinesiology of the performers and not so much on the digital character on the screen. However, there was one participant that paid attention mostly to the digital character and not to the performers. The shape of the character, with the long thin legs gave a sense of fragility, which was in agreement with the creator’s intention. The term “weak” was assigned to the centaur in the performance media by another participant as well, while in the animation they perceived him as “dynamic”, probably due to the use of the camera and the environment (according to the participant).

One participant noted that the performance awoke stronger emotions compared with the animation. Moreover, some participants claimed that in the animation, there was a narration and storyline which helped the perception of the character, while in the performance they missed that. Overall, the majority answered that perceiving the centaur’s (man with tall legs) personality was easier in the animation, because of the narration and the visible facial characteristics and expressions.

For the question “How was birds’ characters’ personality perceived throughout the three media, animation, performance and games”, the majority of the participants mentioned that it was hard for them to spot the personality in the birds, although they enjoyed their movement and presence. Two participants stated that they could distinguish the bird’s personality more easily in the animation because it was a single entity and because there was a story. However, two other participants said that the personality of the birds was more intense in the game due to the interaction that the flock had with the character and the plot.

An interesting topic brought up by one participant was that animals are associated subconsciously with a certain personality, for example, a tiger is fierce, therefore, we might be biased when seeing them and it is harder to identify a different personality

Sounds existed in the live coding and performance formats. As for the live coding, it was clearly hard for the majority of the participants to identify the personality of the birds. Another participant said that the sounds seemed too artificial and that such experimental works need further investigation and the fact that the live coding demo lacked narration or a storyline made things harder. However, sound was appreciated, in general, but as a complementary element to the existing narration. Some participants noticed specifically the differences in sound generation through the wings and through the core, which was also the intention of the creators; each body part would affect the sound in a different manner. However, although most of the participants found the sounds complementary, interesting, and noticed the special connection between movements and sounds, it seems that more elements need to be involved to allow sound to act as a personality indicator.

Two participants brought up the very interesting topic of player’s/user’s personality in terms of the perception of the digital character’s personality. “You might be doing everything right in technical terms but the viewer might perceive a different trait than you (the creator) had in mind!” says one participant, while a third participant continues “ The first time I played the game I was very confused and this affected my perception of the centaur, I thought he was weak and afraid, while when I played the game again and felt confident I thought the character was more dynamic”.

Another interesting issue mentioned by six participants is the inability to simultaneously follow all elements of the performance. Since the performance included voice narration, physical performers moving, one digital character, changes in the environment, and ambient sounds, it seems that it was overwhelming for the participants to follow all these elements. Most stated that they completely missed the narration because they were absorbed in the performer’s movement or the scene.

## 5. Discussion

This discussion section examines how digital character personality was expressed and perceived across four media: animation, games, interactive performance, and interactive networked environments. Each medium afforded different strengths—animation with narrative clarity, games with interactivity, performance with embodied presence, and live coding with experimental dynamics. By comparing these contexts, we highlight how movement, narration, sound, and interaction differently shaped the audience’s interpretation of personality traits. The findings illustrate these differences, showing how participants perceived and evaluated the characters across each demo.

The findings highlight distinct patterns in how the characters in the animation demo were perceived. The male character was initially described by the scriptwriter as sensitive, scared, and weak, and was similarly associated with fear by the participants, while the female character was described as independent, decisive, confident, and strong, and was indeed recognized by the audience as independent and strong. The bird character stood out as the strongest, perceived as independent, decisive, and powerful. Across all elements of this demo, the participants emphasized the importance of movement and interaction as central to the effectiveness of the demonstration, while environmental aspects, such as objects, lighting, and shadowing, were considered less important. Open-ended responses further indicated that features such as gaze, camera focus, background story, music, and dialog could enhance narrative depth and emotional connection. Overall, the results suggest that strong character action combined with movement and interaction are key factors for audience engagement, while additional narrative and cinematic tools could further enrich the experience.

In the game demo, the male character was successfully perceived as independent, decisive, and confident, which was in full agreement with the associations of the script. The female character, who was intended to be sensitive, fearful, and weak, was also largely accepted, and was associated by the participants with adjectives such as sensitivity and fear. The character of the flock of birds was initially identified as a character with self-confidence; the participants largely agreed but also considered him to be independent, decisive, strong, and aggressive. In terms of design elements, all aspects of the Wheel used in the demo were effectively recognized by the participants, with a particular emphasis on movement, interaction, and character appearance characteristics, while the elements related to the environment were considered somewhat less central. The participants noted that adding elements such as camera direction, narration, music, and character conversations would help and enhance the game experience.

In the interactive networked environment demo, the birds were perceived as being mostly independent, confident, determined, and strong. The participants identified body movement most strongly as a defining element of the demonstration, while body and facial features, along with speech and sounds, also contributed to expressing the birds’ personality. Environmental sounds and music were noted as important additions that enriched the overall experience. Movement was rated as the most important category of the Wheel, and participants also reported that the demo’s depiction could be improved by extending immersion through tools such as a 360-degree field of view, more opportunities for interaction in realistic environments, and by using alternative performance styles for the characters.

Other interesting findings indicate that narration and the visibility of facial expressions are crucial for understanding personality, with animation offering clearer perception compared with other formats. In contrast, performance, due to the absence of facial detail and the simultaneous presence of multiple elements (movement, sound, narration, digital environment), often led to a sense of cognitive overload and reduced the clarity in character interpretation.

The birds as characters were mainly evaluated through their movement and interaction with the environment or the player. Their personality was perceived more strongly in the game, due to interactivity, and in animation, due to the narrative framework. In live coding, again, movement was the leading element while sound was less associated. However, stereotypical associations with animal traits (e.g., a tiger as fierce) also influenced participants’ perceptions, shaping how non-human characters were understood. Sound was generally appreciated as a complementary element that enhanced the connection between movement and character, but it was not sufficient on its own to convey personality traits. The experimental nature of live coding, combined with the lack of narration, made it particularly challenging for participants to interpret character identity.

A particularly interesting finding concerned the influence of the viewer’s/player’s own personality on their perception of the digital character. For example, within the game, participants’ feelings of confidence or confusion directly shaped whether they interpreted the centaur as “dynamic” or “weak.” This points to the reciprocal relationship between creator, medium, and audience.

Finally, from the comparison between the designer ‘s intention and the audience perception, the high recognition and agreement observed in the game and interactive network environment underscores the importance of feedback loops, user action, and multimodal cues in audience perception. In contrast, the lower consistency in animation demonstrates that non-interactive, purely visual expression alone is not sufficient to effectively convey complex personality traits. These findings validate the theoretical framework underlying the Taxonomy Wheel and highlight the importance of interaction in digital arts.

Overall, this study demonstrates that the perception of personality does not depend solely on the medium itself but rather on a combination of factors: narration, the visibility of expressions, kinesiology, sound, directorial guidance, and the viewer’s own experience and disposition. Future research could benefit from more controlled comparisons, applying identical narrative and audio–visual conditions across different media to better isolate the effect of the medium. Additionally, greater public familiarization with experimental formats such as live coding will be essential to fully explore their potential for character development.

### Limitations

While this study provides valuable insights into how digital character personality can be conveyed across multiple media, several limitations should be acknowledged. First, the sample size of 14 participants, though diverse in disciplinary backgrounds, limits the generalizability of the findings. A larger and more demographically varied participant pool would allow for stronger statistical conclusions and better capture the cultural or contextual differences in personality perception. Second, the experimental conditions differed slightly between media, making it difficult to fully isolate the influence of each modality (animation, game, performance, and live coding). Factors such as narration, sound design, or the degree of interactivity were not held constant across prototypes, which may have affected the audience’s interpretation of character traits. Finally, subjective interpretation remains a challenge: participants’ personal moods, familiarity with the media, and even their own personality traits influenced perception, as acknowledged in several interview responses. This variability underscores the complexity of assessing personality perception as a cross-media construct.

## 6. Conclusions and Future Work

This study investigated how motion capture and biosignals can be integrated into character workflows across animation, games, installations, and live performance. By developing four prototypes, we proposed and validated a workflow for augmenting the personality expression of digital characters through sensor-based technologies. The findings show that personality perception emerges from the interplay of movement, interaction, narration, sound, and audience disposition, rather than from any single medium or tool. Beyond the specific case studies, this research offers methodological insights and technical tools for designing more expressive and emotionally resonant digital characters in trans-media contexts.

Future research will aim to address the identified limitations by developing more controlled cross-media experiments, in which identical narrative and audio–visual elements are implemented across different formats. This approach will enable the clearer identification of observed differences in the characteristics of each medium. In addition, this study could be extended to other emerging media and technologies such as virtual and mixed reality environments, where embodied interaction, sensory immersion, and spatial presence may further influence the perception of character personality. Finally, particular attention should be given to more experimental media, including interactive networked environments and interactive performances, which are not yet as established or standardized as animation and games. Future work should focus on refining the associated workflows, interaction models, and technical functionalities to enhance their expressive potential and accessibility for creators.

## Figures and Tables

**Table 1:** Animation demo characters and their personality characteristics.

Demos/Designer’s Intentions, Characteristics	Animation
Taxonomy Wheel	Body movement, lighting and shading, scene objects, character–character interaction, body and facial characteristics
Characters	Male character—Dali Female character—turtle Bird
Personality (according to creator)	Male character—Dali: sensitive, scared, weak Female character—turtle: independent, determined, confident, dominant Bird: aggressive

**Table 2:** Game demo characters and their personality characteristics.

Demos	Demo II—Video Game
Taxonomy Wheel	Body movement, lighting and shading, scene objects, character–character interaction, character–environment interaction, body and facial characteristics
Characters	Male character—centaur Female character—centaur Flock of birds
Personality according to creator	Male character—centaur: independent, determined, confident Female character—centaur: sensitive, scared, weak Flock of birds: confident

**Table 3:** Interactive performance demo characters and their personality characteristics.

Demos	Demo IV—Interactive Performance
Taxonomy Wheel	Body movement, lighting and shading, ambient sounds, scene objects, body and facial characteristics, voice
Characters	Male character—Dali Performers
Personality according to creator	Male character—Dali: independent, confident, strong

**Table 4:** Interactive network environment demo characters and their personality characteristics.

Demos	Demo III—Interactive Network Environment
Taxonomy Wheel	Body movement, ambient sound, body and facial characteristics
Characters	2 birds
Personality according to creator	Birds: independent, confident, strong

**Table 5:** Connection between sensors used and the taxonomy Wheel aspects.

Sensor Type	Mapped Aspect on the Wheel	Main Personality/Emotion Influence
Motion Capture	Motion–Action, Interaction	Extraversion, Openness
ECF/HRV	Motion–Action, Environment	Emotional Stability, Arousal
EDA/GSR	Structure, Environment	Arousal Intensity
EMG	Structure, Motion–Action	Valence
Temperature	Structure, Environment	Trust–Disgust, Calm–Fear
Respiration	Motion–Action, Environment	Anxiety–Relaxation
Sonification	Environment, Interaction	Emotional Resonance, Empathy

**Table 6:** Custom traits, their theoretical foundations, and core interpretations as defined by the creative team.

Trait	Related Models	Core Interpretation
Independent	Big Five—Conscientiousness, Extraversion; MBTI archetypes	Leadership, autonomy, and initiative; self-discipline and agency
Decisive	Big Five—Conscientiousness, Extraversion; MBTI (ENTJ “Commander”); CSI—Preciseness	Assertive choices, clarity, goal-directed behavior; determination in action and communication
Self-confident	Big Five—low Neuroticism; Realact; LMA (free, expansive gestures)	Emotional stability, resilience, assurance; openness expressed in posture and gesture
Strong	Big Five—Extraversion + Emotional Stability; MBTI (ENTJ “Commander”); Realact; LMA (strong weight, expansive space)	Physical power, dominance, assertive presence; embodied through expansive, forceful movement
Sensitive	Big Five—high Neuroticism + Agreeableness; MBTI (INFP “Mediator”); OCC (appraisals of others); CSI—Emotionality	Empathy, vulnerability, attentiveness to others; affect-laden interaction
Weak	Big Five—low Extraversion, low Conscientiousness; Realact; LMA (light, bound qualities)	Passivity, fragility, withdrawal; embodied in hesitant, constrained movement
Fearful	Big Five—high Neuroticism; MBTI (INFP “Mediator”); OCC (threat/loss appraisals); LMA (bound, hesitant flow)	Anxiety, avoidance, cautiousness; shrinking posture and inhibited actions
Aggressive	Big Five—low Agreeableness; OCC (hostility appraisals); Realact (over-modulated behavior); CSI—Verbal Aggressiveness	Dominance, hostility, antagonism; conveyed through forceful and abrupt movements

## Data Availability

The original contributions presented in this study are included in the article. Further inquiries can be directed to the corresponding author.
